# Acute kidney injury in diabetic patients: A narrative review

**DOI:** 10.1097/MD.0000000000033888

**Published:** 2023-05-26

**Authors:** Amninder Kaur, Gaurav Shekhar Sharma, Damodar R Kumbala

**Affiliations:** a Senior Resident, Department of Nephrology, All India Institute of Medical Sciences Rishikesh, Uttarakhand, India; b Assistant Professor, Department of Nephrology, All India Institute of Medical Sciences Rishikesh, Uttrakhand, India; c Diagnostic and Interventional Nephrologist, Renal Associates of Baton Rogue, Baton Rogue, LA.

**Keywords:** diabetes mellitus, acute kidney injury, diabetic kidney disease, chronic kidney disease

## Abstract

Diabetes mellitus (DM) is the most common cause of chronic kidney disease, which leads to end-stage renal failure worldwide. Glomerular damage, renal arteriosclerosis, and atherosclerosis are the contributing factors in diabetic patients, leading to the progression of kidney damage. Diabetes is a distinct risk factor for acute kidney injury (AKI) and AKI is associated with faster advancement of renal disease in patients with diabetes. The long-term consequences of AKI include the development of end-stage renal disease, higher cardiovascular and cerebral events, poor quality of life, and high morbidity and mortality. In general, not many studies discussed extensively “AKI in DM.” Moreover, articles addressing this topic are scarce. It is also important to know the cause of AKI in diabetic patients so that timely intervention and preventive strategies can be implemented to decrease kidney injury. Aim of this review article is to address the epidemiology of AKI, its risk factors, different pathophysiological mechanisms, how AKI differs between diabetic and nondiabetic patients and its preventive and therapeutic implications in diabetics. The increasing occurrence and prevalence of AKI and DM, as well as other pertinent issues, motivated us to address this topic.

## 1. Introduction

Type 2 diabetes is one of the primary causes of chronic kidney disease (CKD) and end-stage renal disease (ESRD) globally.^[1]^ Over the past 2 decades, diabetes mellitus incidence and prevalence have steadily increased, from an estimated 30 million in 1985 to 537 million in 2021 and it is anticipated that the number will increase to 643 million by 2030 and 783 million by 2045.^[2]^ Considering recent trends, microvascular and macrovascular disorders are associated with long-term diabetes-related complications. One significant microvascular sequelae of diabetes are diabetic nephropathy (DN) or diabetic kidney disease (DKD).^[3]^ Around 20% to 40% of diabetic patients are thought to suffer from DKD, and approximately 40% will need renal replacement therapy at some point in their lifetime.^[4,5]^ Risk factors for the progression of CKD include gender, racial disparities, hereditary factors, concurrent comorbid illnesses, for example, diabetes mellitus (DM), metabolic abnormalities, and previous episodes of acute kidney injury (AKI), etc.^[6]^

AKI, which affects up to 1% of the general population and 15% of all hospitalized patients, is a worldwide health issue.^[7–9]^ Diabetes is a distinct risk factor for AKI.^[10]^ Although baseline DM is also an independent risk factor for AKI in multivariate analyses adjusted for estimated glomerular filtration rate (eGFR).^[11]^ There have been other studies that showed that patients with diabetes may be more prone to AKI.^[12,13]^ Acute tubular injury caused by renal insults may have an impact on kidney function, leading to chronic functional impairment and later maladaptive recovery and failure to entirely undo the insults.^[14,15]^ Generally, there is a significant correlation between AKI and the emergence of CKD and ESRD.^[16,17]^ In a cohort of 4082 patients with diabetes, Thakar et al^[18]^ showed that AKI episodes were related to a cumulative likelihood of developing progressive CKD, regardless of the presence of any other significant risk factors for progression. Subsequently, a large prospective study provided additional evidence that AKI is a strong indicator of unfavorable outcomes (doubling of serum creatinine or ESRD) and mortality in diabetes.^[19]^

## 2. Epidemiology of AKI in diabetes mellitus

Girman et al^[20]^ compared 119,966 diabetic patients with 1794,516 nondiabetic patients and showed that the incidence of AKI was significantly higher in diabetic patients (198 per 100,000 person-years vs 27 per 100,000 patient-years; crude hazard ratio, 8.0; 95% confidence interval, 7.4–8.7). Despite accounting for additional known comorbidities and AKI risk factors, the differences remained statistically significant. However, in their study, a clinical coding system rather than a biochemical definition of AKI was employed, which could result in significant under-ascertainment. Additionally, a meta-analysis by James et al revealed that participants with diabetes had higher AKI hazard ratios than participants without diabetes, regardless of their eGFR levels. Again, the AKI definition in these studies was based on administrative codes, which underestimated mild forms of AKI.^[21]^

In a retrospective cohort study by Prabhu et al^[22]^, an annual AKI incidence was 12.6%. There was a substantial deviation from previously published studies by Thakar et al^[18]^ and Monseu et al^[19]^, who reported AKI incidence of 2.8% and 5.2%, respectively.

In their retrospective cohort of 16,700 participants (9417 with type 2 diabetes and 7283 nondiabetic controls), Hapca et al^[23]^ found that diabetic patients had higher rates of AKI than controls (48.6% vs 17.2%, respectively). The AKI risk among diabetic patients was 5 times higher than that of controls, even in the absence of CKD (121.5 vs 24.6 per 1000 person-years). AKI rates in diabetic patients with CKD were twice as high as in controls (384.8 vs 180.0 per 1000 person-years after the onset of CKD, and 109.3 vs 47.4 per 1000 person-years before the onset of CKD).

Recently, Venot et al^[24]^ in their prospective case-control study, which included patients with severe sepsis and septic shock with or without diabetes, found that the incidence of AKI did not differ between the 2 groups however, diabetic patients requiring dialysis more often, had higher mean serum creatinine levels, and less recovery than nondiabetic patients. However, this study has several limitations, as the diagnosis was made based on the medical history, long-term diabetic complications, and HBA1C levels were not incorporated, initial renal function status was missing, and for the diagnosis of AKI, urine output criteria were not used. Finally, the requirement for dialysis was not assessed using defined criteria. This absence of differences between these 2 groups may be explained by these confounding factors.

Very few studies have examined concurrent AKI, CKD, and recurrent AKI in this group of patients.^[18]^ Table 1 summarizes studies related to this topic.

**Table 1:** Few studies assessed AKI incidences and outcomes in diabetic patients.

Study	Design	Sample size (n)	Results
Mehta et al 2006^[25]^	Retrospective data analysis	449,524 Prevalence of Diabetes mellitus in patients with AKI after cardiac surgery	DM prevalence is high in AKI vs no AKI (49 vs 33%) (*P* < .0001)
Mittalhenkle et al 2008^[26]^	Prospective case-control study	5731 AKI incidence in the elderly population	Diabetes mellitus associated with incident AKI
Oliveira et al 2009^[27]^	Prospective, single center	980 Prevalence of DM in aminoglycoside-induced AKI	High DM prevalence in AKI vs no AKI (19.6 vs 9.3%) (*P* < .007)
Orban et al 2014^[28]^	Retrospective data analysis, single center analysis	94 Risk factor of AKI on ICU admission	High blood glucose associated with increased occurrence of AKI on ICU admission
Venot et al 2015^[24]^	Prospective case-control study	318 diabetic vs 746 nondiabetic AKI incidence and outcomes in diabetic vs nondiabetic patients with severe sepsis/septic shock	AKI incidence is not different Higher serum creatinine and dialysis frequency in diabetic patients
Kim et al 2019^[29]^	Case-matched retrospective analysis	884 Effect of DM on AKI after minimally invasive partial nephrectomy	The incidence of postoperative AKI in DM vs non-DM patients was 30.7 vs 14.9% (*P* < .001)

AKI = acute kidney injury, DM = diabetes mellitus.

## 3. Risk factors for AKI

### 3.1. Diabetes and non-modifiable factors

Girman et al^[20]^ reported that diabetes alone was still associated with a higher risk of acute kidney failure, even after accounting for other risk factors, such as chronic kidney disease. Additional risk factors for AKI were increasing age, chronic kidney disease, systemic hypertension, previous history of AKI, and congestive cardiac failure. The combination of type 2 diabetes with congestive cardiac failure or systemic hypertension further increases the risk of AKI. In a previous study, elderly patients with heart failure had a 3.37-fold higher risk of AKI than those without heart failure, whereas hypertension was linked to a 1.94-fold higher risk of acute kidney injury.^[30]^ Acute kidney injury occurred in 21% of patients in a cohort study admitted with congestive heart failure as their primary diagnosis.^[31]^ preexisting proteinuria, hypertension, and diabetes mellitus were all independent AKI risk variables revealed in the study by Hsu et al^[11]^

### 3.2. Proteinuria and lower eGFR

According to Prabhu et al^[22]^, there was a correlation between AKI incidence and lower baseline eGFR, and higher proteinuria. With every 1 g rise in proteinuria, they have revealed that AKI risk was increased by 15.8%. Moseu et al came to the same conclusion regarding the correlation of AKI incidence with lower eGFR and albuminuria.^[19]^ A large cohort study showed moderate to high proteinuria was a risk factor for AKI among all eGFR groups in hospitalized patients with AKI.^[32]^

### 3.3. Hypoglycemic agents

In comparison, nondiabetic patients, surgical patients with diabetes, and those taking oral antidiabetic medications had a 30% higher chance of developing acute renal failure following surgery, while those taking insulin had a 70% higher risk.^[33]^

### 3.4. Drugs

Drug-induced AKI represents 20% of all etiologies.^[34]^ Patho physiological mechanism depends on the type of drug involved.^[34]^ ACE/ARB (angiotensin-converting enzyme/angiotensin receptor blocker) were the main causes of AKI contributing to 35% of cases due to their increased use in diabetic patients. This risk was even higher in patients with congestive heart failure, volume depletion, diuretics, nonsteroidal anti-inflammatory drugs (NSAIDs), and bilateral renal artery stenosis. Aminoglycoside (gentamicin) and NSAIDs contributed to 16% followed by statins (10%), antitubercular agents (rifampicin), and ifosfamide 6% and 3% respectively. Glomerular filtration rate and renal blood flow are decreased as a result of the suppression of prostaglandin production due to NSAIDs. They also reported dehydration and intravenous rehydration as prognostic factors in their study.^[35]^

The histological lesion of AKI caused by diuretics may primarily manifest as tubular epithelial cell vacuolation. Risk is even higher in a combination of other drugs that is, NSAIDs, antibiotics, ACEi, and contrast.^[36]^ Diabetic patients who used hydrochlorothiazide (HCT) frequently experienced renal events (decline in eGFR > 30%), which affected about 20% of individuals as shown in a retrospective study.^[37]^ Similarly, another study showed that diuretic-associated AKI patients had a higher rate of comorbidities (DM, CVD, CKD, hypertension) as compared to the non-diuretic AKI group. In the diuretic-induced AKI group, 27.5% was caused by diuretics only and 29.8% was caused by the combination of diuretics with other drugs.^[36]^

AKI hospitalizations in the US have recently increased considerably, from 35,000 in 1979 to 650,000 in 2002. This increase was attributed to the increasing drug consumption by the elderly, and various comorbidities.^[38]^ Similarly, from 1992 to 2001, AKI incidence among Medicare beneficiaries increased by 11% annually, with higher rates seen in the elderly, men, and African-Americans.^[39]^

Although it is believed that the use of ACE/ARB is linked to acute renal failure, it can be challenging to interpret published studies because those who are most at risk for AKI may also be the ones who are most likely to receive treatment with angiotensin-converting enzyme/ARBs.^[40]^

### 3.5. Dehydration

Extracellular volume depletion due to glycosuria because of uncontrolled diabetes especially in pediatric patients leads to prerenal AKI.^[41]^ The combined effect of uncontrolled diabetes along with prerenal AKI may cause intrinsic renal AKI, characterized by renal parenchymal damage and tubular necrosis.^[42]^

### 3.6. Sepsis

Along with dysfunctional immune systems both humoral and cell-mediated, increase neutrophil dysfunction also contributes to an increased risk of sepsis.^[22]^ Sepsis was the primary cause of AKI in a retrospective study done by Prabhu et al^[22]^ In addition, they showed that there was a higher eGFR decline secondary to sepsis-related AKI as compared to other etiologies. Diabetes mellitus has been demonstrated to be an independent risk factor in a recent meta-analysis of sepsis-related AKI.^[43]^

### 3.7. Contrast

Diabetic patients, particularly those who developed DN, are more prone to contrast-induced injury. Diabetes and contrast-induced acute kidney injury (CI-AKI) are mutually causative, causing kidney function to deteriorate further. Renal hypoxia, generation of reactive oxygen species, and increased oxidative stress in diabetic patients lead to vascular constriction due to vasoactive substances. Immunological changes in diabetic patients also contribute to contrast-induced AKI. Signaling pathways that is, inflammation, reactive oxygen species production, and apoptosis related to both diabetes and contrast-induced AKI.^[44]^ Due to impaired nitro vasodilation, increased endothelin synthesis, and hyperresponsiveness to adenosine-related vasoconstriction, peritubular blood flow may also be affected.^[45]^ According to data, the incidence of Contrast-induced AKI ranges from 5.7% to 29.4% in diabetes patients and is approximately 13% in nondiabetic patients.^[46]^ A recent meta-analysis showed that diabetes is associated with a higher risk of CI-AKI. Moreover, the subgroup of DM patients with CKD had a greater predictive effect of elevated CI-AKI but this correlation was not significant in the subgroup of patients without CKD.^[47]^

Table 2 summarizes the causes of AKI in diabetic patients.

**Table 2:** Risk factors of AKI in diabetic patients.

Susceptibilities	Exposures
Diabetes	Nephrotoxic drugs: NSAIDs, ACEi, contrast, antibiotics, diuretics
Advanced age	Sepsis/septic shock
Chronic kidney disease	Surgery
Systemic hypertension	Dehydration
Congestive cardiac failure history	
Previous episode of AKI	
Pre-existing proteinuria	

AKI = acute kidney injury, NSAIDs = nonsteroidal anti-inflammatory drugs.

## 4. Pathophysiologic mechanisms of AKI in patients with diabetes

**Figure 1.**
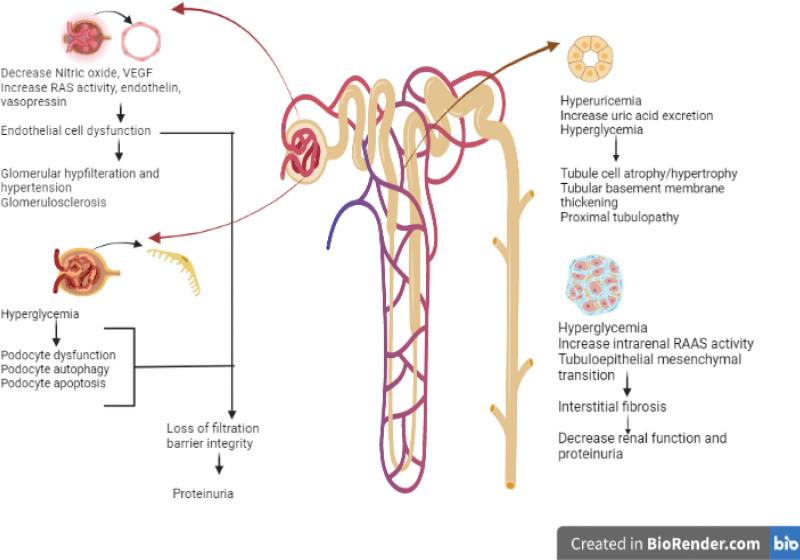
Pathophysiological mechanism of diabetes-induced kidney damage.

The pathophysiologic mechanisms causing diabetes-related kidney damage are multifactorial. (Fig. 1) It has been hypothesized that structural and functional alterations in the renal vasculature and the tubular epithelial cells increase the cytokines and chemokines generation, which produce inflammation, ischemia, and isolated proximal tubulopathy.^[48,49]^

Endothelial cell dysfunction is one of the main mechanisms underlying DN. Diabetic kidneys are known to produce less nitric oxide (NO), which is produced by the enzyme endothelial nitric oxide synthase (eNOS). Because of diabetes’s distorted NO metabolism, the renal vasculature is more vulnerable to stimuli that cause vasoconstriction.^[3]^

It is believed that in uncontrolled diabetes, renal vasculature dysregulation is the primary factor contributing to glomerular hyperfiltration.^[50,51]^ Persistent glomerular hyperfiltration causes intraglomerular hypertension, followed by glomerulosclerosis, which causes a progressive decline in kidney function and eventually DKD.^[50,51]^

In the case of prerenal AKI, when the body depends on variations in renal vascular resistance to maintain blood pressure, a dysregulation in normal renal vascular tone may hasten the kidney damage.^[52,53]^ The absence of a suitable vascular counterregulatory response to sustain kidney blood flow can also significantly worsen kidney hypoperfusion.^[50,51]^

Chronic and acute renal damage related to diabetes may be exacerbated by hyperuricemia.^[54]^ It has been demonstrated that hyperuricemia can cause crystal-mediated and crystal-independent nephropathy, glomerular injury, and tubulointerstitial involvement.^[55]^ It is crucial to remember that hyperuricemia could signify dehydration, which can directly cause renal injury.

Persistent hyperglycemia, which is related to prolonged ICU stays and an increased risk of AKI is another pathophysiologic pathway that results in CKD and eventually ESRD.^[3]^ Apoptosis of endothelial cells, vascular rarefaction and hypoxia, mitochondrial dysfunction, proximal tubular disorder, podocyte disorder, podocyte apoptosis, and autophagy due to diabetes have all been shown in laboratory studies.^[3]^

## 5. AKI in diabetic versus nondiabetic patients

Diabetic patients are at higher risk of AKI than nondiabetic patients, which can be attributed to diabetes, chronic kidney disease, hyperglycemic crisis, drugs that is, ACE inhibitors and sodium-glucose cotransporter-2 (SGLT2) inhibitors, associated cardiovascular disease and heart failure, and previous AKI episodes.^[56]^ Girman et al^[20]^ in a retrospective cohort showed that diabetic patients were 8 times more likely to have incident acute renal failure than nondiabetic patients. There have been 2 retrospective analyses, both of which had conflicting findings.^[10,57]^ Diabetic patients had less severe AKI, recovery to baseline renal function and the proportion of patients developing progressive CKD was lower in the diabetic group as shown by Johns et al^[10]^ However, the 10-year retrospective analysis done by Xin S et al showed that the recurrence rate of AKI was higher in the diabetic group than in the nondiabetic group.^[57]^ Between diabetes and nondiabetic groups, mortality was comparable in both retrospective analyses.

## 6. Diabetes and cardiorenal syndrome

Cardiorenal syndrome (CRS) is a disease affecting the heart and kidneys simultaneously. T2DM is a significant risk factor for the development of CRS; the National Health and Nutrition Examination Survey in the USA found a strong association between type 2 CRS and T2DM.^[58]^ Moreover, being a systemic disorder affecting the heart and kidneys, and it is also associated with type 4 and type 5 CRS.^[59]^ SGLT 2 inhibitors were implicated in CRS due to both renal and cardioprotective effects. Reno protective effects of SGLT2 inhibitors in AKI contributed by increased vascular endothelial growth factor A expression, increase vasodilatation due to NO, and decrease renal fibrosis.^[60]^ Regardless of the presence of atherosclerotic CVD or a history of heart failure, these drugs decrease the hospitalization rate for heart failure and the progression of renal illness.^[61]^ EMPA-REG OUTCOME (Empagliflozin cardiovascular outcome event trial in type 2 diabetes mellitus), DECLARE-TIMI 58, CANVAS (Canagliflozin Cardiovascular Assessment Study), and CREDENCE (Canagliflozin and Renal Events in Diabetes with Established Nephropathy) are the 4 major trials in diabetic patients showed positive cardiovascular and renal outcomes.^[62–65]^ In December 2016 there was an FDA alert regarding the use of canagliflozin and dapagliflozin.^[66]^ However, this increased risk of AKI with SGLT2 inhibitors was not supported by studies.^[67]^ Meta-analysis showed the protective effect of SGLT2 inhibitors with AKI, primarily driven by empagliflozin.^[68]^

These drugs should not be started in CRS 1 and 3 but may be continued with close hemodynamic and renal function monitoring. SGLT2 inhibitors are preferred drugs in CRS types 2, 4, and 5 for glycaemic, as well as metabolic, control.^[69]^

## 7. Preventive strategies and implications

In AKI, there is no universal therapy for AKI. The primary goals of treatment are to address underlying causes, such as dehydration, avoiding nephrotoxic drugs, fluid, and electrolyte management, and renal replacement therapy.^[70]^

Obese patients have glomerulomegaly, increase renal blood flow, hyperfiltration, and higher albuminuria despite the absence of hypertension.^[71]^ Moreover, sleep apnea in obese patients causes hypoxic episodes contributing to renal impairment.^[72]^ Hence weight control is an important preventive aspect in terms of decreasing renal injury.

Renin-angiotensin-aldosterone system inhibitors have been shown to attenuate proteinuria and continue to be the cornerstone of current therapeutic methods.^[73,74]^ As patients with T2DM has more significantly greater urinary albumin-to-creatinine ratios than patients with T1DM even after adjustment for all known risk factors for diabetic kidney disease, as shown in the SEARCH study, hence RASS inhibitors are indicated in diabetic patients treatment frequently.^[75]^ However, these drugs have nephrotoxic effects directly or indirectly by affecting renal hemodynamic function.^[72,74]^

Many antihyperglycemic agents, including metformin, thiazolidinediones, dipeptidyl peptidase inhibitors, Glucagon-like peptide agonists, and SGLT2 (Sodium-glucose Cotransporter-2) inhibitors, also have nephroprotective properties in addition to glucose-lowering effects.^[76–78]^ However, accumulation of metformin in case of impaired eGFR (e.g., 30–60 mL/minutes/1.73 m2) causes type B lactic acidosis and toxicity by impairing mitochondrial function. Thiazolidinediones, such as pioglitazone, have been shown to decrease proteinuria in a large meta-analysis.^[46]^ However, no randomized controlled trials showed renal protective effects of thiazolidinediones.^[79]^ Role of dipeptidyl peptidase inhibitors and glucagon-like peptide 1 receptor agonist as nephroprotective agents are controversial. These have been shown to have nephroprotective effects in some studies.^[76,80]^ However their effects on eGFR were uncertain as shown in a recent Cochrane review.^[80]^ SGLT2 inhibitors were found to have a nephroprotective effect as shown in DECLARE-TIMI 58 and EMPA-REG OUTCOME trials, but in the Cochrane review, it did not show any effect on AKI risk.^[80–82]^

Some drugs have been evaluated in animal models and may represent future therapeutic options for AKI prevention, such as mineralocorticoid receptor antagonists, endothelin receptor antagonists, peroxisome proliferator-activated receptors agonists, and phosphodiesterase inhibitors. In animal models, finerenone also decreases the progression of AKI to CKD, and hence it can be an excellent therapeutic option in AKI to prevent long-term complications.^[83]^

## 8. Conclusion

In summary, AKI is a complication of diabetes mellitus. It increases the risk of further episodes of AKI, progression to chronic kidney disease, end-stage renal disease, cardiac and cerebrovascular events, and all-cause morbidity and mortality. Additionally, diabetes is the risk factor for AKI irrespective of underlying CKD. Current strategies should focus on its identification and mitigation, reducing proteinuria, weight control, fluid management, removal of precipitant factors (drugs, sepsis, contrast), and other supportive measures to improve AKI outcomes. Many glucose-lowering drugs (SGLT2 inhibitors) have nephroprotective against AKI in patients with diabetes in addition to their antidiabetic effects. A promising new approach to treating AKI and CKD using novel classes of medications that target renal hemodynamic dysfunction in diabetic patients. In the interim, healthcare professionals need to be aware of the risks and effects of AKI in patients with diabetes.

## Author contributions

**Conceptualization:** Amninder Kaur, Gaurav Shekhar Sharma, Damodar Kumbala.

**Supervision:** Gaurav Shekhar Sharma.

**Validation:** Gaurav Shekhar Sharma, Damodar Kumbala.

**Visualization:** Amninder Kaur, Damodar Kumbala.

**Writing – original draft:** Amninder Kaur.

**Writing – review & editing:** Amninder Kaur, Gaurav Shekhar Sharma.s
